# Screening for BRCA2 mutations in 81 Dutch breast–ovarian cancer families

**DOI:** 10.1054/bjoc.1999.0892

**Published:** 1999-12-08

**Authors:** T Peelen, M van Vliet, A Bosch, G Bignell, H F A Vasen, J G M Klijn, H Meijers-Heijboer, M Stratton, G-J B van Ommen, C J Cornelisse, P Devilee

**Affiliations:** Departments of Human Genetics, Pathology, Foundation for the Detection of Hereditary Tumors, Leiden University Medical Center, Leiden, The Netherlands; Institute of Cancer Research, Molecular Carcinogenesis Section, 15 Cotswold Road, Belmont, Sutton, Surrey SM2 5PS, UK; Family Cancer Clinic, Dr. Daniel den Hoed Cancer Center and Academic Hospital, Groene Hilledijk 301, Rotterdam, EA 3075, The Netherlands; Department of Clinical Genetics, Erasmus University, Westzeedijk 112, P.O. Box 1783, Rotterdam, DR 3000, The Netherlands

**Keywords:** BRCA1, BRCA2, mutation analysis, breast cancer

## Abstract

We have analysed 81 families with a history of breast and/or ovarian cancer for the presence of germline mutations in BRCA2 with a number of different mutation screening techniques. The protein truncation test (PTT) for exons 10 and 11 detected four different frame-shifting mutations in six of these families. Four of the remaining 75 families had given positive linkage evidence for being due to BRCA2. In these families the entire coding region was analysed by single-strand conformational polymorphism, leading to the detection of a non-sense and a splice-site mutation in two of them. While these studies were in progress, Southern analysis of BRCA1 revealed that in our study-population of 81 families, 15 families were segregating either the exon 13 or exon 22 deletion in BRCA1 (Petrij-Bosch et al (1997) *Nat Genet***17**: 341–345). This prompted us to examine BRCA2 in the remaining 58 families by Southern analysis, using two different restriction enzymes. No aberrations were found in the restriction patterns. Thus, contrary to BRCA1, large genomic rearrangements within the BRCA2 gene do not represent a major mutation mechanism among Dutch breast cancer families. © 2000 Cancer Research Campaign


*British Journal of Cancer* (2000) **82**(1), 151–156
© 2000 Cancer Research Campaign
Article no. bjoc.1999.0892

### Screening for BRCA2 mutations in 81 Dutch breast–ovarian cancer families

**T Peelen[1], M van Vliet[1,6], A Bosch[1], G Bignell[4], HFA Vasen[3], JGM Klijn[5], H Meijers-Heijboer[6], M Stratton[4], G-JB van Ommen[1], CJ Cornelisse[2] and P Devilee[1,2]**

Departments of [1]Human Genetics and [2]Pathology and [3]Foundation for the Detection of Hereditary Tumors, Leiden University Medical Center, Leiden, The Netherlands; [4]Institute of Cancer Research, Molecular Carcinogenesis Section, 15 Cotswold Road, Belmont, Sutton, Surrey SM2 5PS, UK; [5]Family Cancer Clinic, Dr. Daniel den Hoed Cancer Center and Academic Hospital, Groene Hilledijk 301, 3075 EA Rotterdam, The Netherlands; [6]Department of Clinical Genetics, Erasmus University, Westzeedijk 112, P.O. Box 1783, 3000 DR Rotterdam, The Netherlands

**Summary** We have analysed 81 families with a history of breast and/or ovarian cancer for the presence of germline mutations in BRCA2 with a number of different mutation screening techniques. The protein truncation test (PTT) for exons 10 and 11 detected four different frame-shifting mutations in six of these families. Four of the remaining 75 families had given positive linkage evidence for being due to BRCA2. In these families the entire coding region was analysed by single-strand conformational polymorphism, leading to the detection of a non-sense and a splice-site mutation in two of them. While these studies were in progress, Southern analysis of BRCA1 revealed that in our study-population of 81 families, 15 families were segregating either the exon 13 or exon 22 deletion in BRCA1 (Petrij-Bosch et al (1997) *Nat Genet* **17**: 341–345). This prompted us to examine BRCA2 in the remaining 58 families by Southern analysis, using two different restriction enzymes. No aberrations were found in the restriction patterns. Thus, contrary to BRCA1, large genomic rearrangements within the BRCA2 gene do not represent a major mutation mechanism among Dutch breast cancer families. © 2000 Cancer Research Campaign

**Keywords:** BRCA1; BRCA2; mutation analysis; breast cancer

Hereditary breast cancer is a genetically and clinically heterogeneous disease, and several susceptibility genes have been identified to date (Rahman and Stratton, 1998). The Breast Cancer Linkage Consortium analysed 237 families with at least four cases of breast cancer and any number of ovarian cancer, and estimated that 81% of the breast–ovarian cancer families were due to BRCA1 on 17q12–21, with most others (14%) due to BRCA2 on 13q12–13 (Ford et al, 1998). Conversely, 76% of the families with male and female breast cancer were due to BRCA2. The largest proportion (67%) of families due to other genes was found in families with four or five cases of female breast cancer only.

The disease associated mutation spectrum of the BRCA1 gene is predominantly composed of small insertions and deletions, leading to premature translation termination (Breast Cancer Information Core (BIC) Website, March 1999). Although these mutations occur throughout the entire coding region, several have been repeatedly detected in specific populations. For most of these mutations, haplotype analyses of the carriers strongly suggested a founder effect (Szabo and King, 1997). For the Dutch population we have also described several founder mutations in the BRCA1 gene. For example, the 2804delAA mutation, constitutes approximately 15% of the total Dutch BRCA1 mutation spectrum (Peelen et al, 1997). Two large genomic deletions, encompassing exons 13 and 22 respectively, were also found to be major founder mutations, accounting for at least 25% of the mutations described in the Dutch population thus far (Petrij-Bosch et al, 1997). These deletions probably arose through Alu-mediated recombination, and are difficult to detect by polymerase chain reaction (PCR)-based mutation screening.

The mutation spectrum of BRCA2 is less well characterized than that of BRCA1, mainly because fewer BRCA2 mutations have been reported to date. By far the greatest majority of them are again frame-shifting (i.e. small deletions and insertions) and non-sense mutations, which occur in all coding exons (Breast Cancer Information Core Website). Few major founder mutations have so far been detected: the 999del5 mutation in the Icelandic population, the 6174delT mutation in the Ashkenazi Jewish population, and the 8765delAG mutation in the French-Canadian population (Neuhausen et al, 1996, 1998; Thorlacius et al, 1996; Tonin et al, 1998). We employed the protein truncation test (PTT) to search for mutations in exons 10 and 11 of the gene, which together comprise 59% of the coding region, in a total of 81 Dutch hereditary breast cancer families. Furthermore, we also applied Southern analysis of the entire coding region of BRCA2, to investigate whether large genomic deletions, like in BRCA1, could form a significant proportion of the Dutch mutation spectrum.

*Received 25 March 1999*
*Revised 22 June 1999*
*Accepted 7 July 1999*

*Correspondence to:* P Devilee, Department of Human Genetics, Leiden University Medical Center, Wassenaarseweg 72, 2333 AL Leiden, The Netherlands

### SUBJECTS AND METHODS

### Family selection

Families were ascertained by the Foundation for Detection of Hereditary Tumors in Leiden and the Family Cancer Clinic and the Department of Clinical Genetics in Rotterdam. Medical and/or

**Table 1** BRCA2 exon 10 and 11 PTT primer sequences

| Primer name | Sequence | Product length |
|---|---|---|
| BR2–10F[a] | 5′ GCT TTA ACA GGA TTT GGA AAA ACA TC 3′ | |
| BR2–10R | 5′ AAA CAC AGA AGG AAT CGT CAT C 3′ | 1169 |
| BR2–11A-F[a] | 5′ GTG CAT TCT TCT GTG AAA AGA AGC 3′ | |
| BR2–11A-R | 5′ GCA CTT CAA ATG TAC TCT TCT GC 3′ | 1517 |
| BR2–11B-F[a] | 5′ GTA AAG CAG CAT ATA AAA ATG ACT C 3′ | |
| BR2–11B-R | 5′ TTT CAT CAC GTT CGG GTT GT 3′ | 1625 |
| BR2–11C-F[a] | 5′ GCT GCT GCC AGT AGA AAT TCT CA 3′ | |
| BR2–11C-R | 5′ TTC GGA GAG ATG ATT TTT GTC A 3′ | 1208 |
| BR2–11D-F[a] | 5′ GAA GAA AGA GCA AAA AGT CCT GC 3′ | |
| BR2–11D-R | 5′ AAA CTT GCT TTC CAC TTG CTG 3′ | 1244 |
| BR2–11E-F[a] | 5′ GCT TGT GGG ATT TTT AGC ACA GC 3′ | |
| BR2–11E-R | 5′ TGG CAA CAC GAA AGG TAA AA 3′ | 932 |

[a] All forward primers carry a Kozak sequence to initiate translation: 5′ CGC TAA TAC GAC TCA CTA TAG GAA CAG ACC ACC ATG 3′.

pathological records verified cancer diagnoses, if available. The families were selected on the following criteria: no BRCA1 mutation at the time they entered the study, and at least two cases of breast cancer diagnosed under 60, or ovarian cancer at any age. When lod-scores had been calculated (Cornelis et al, 1995; Peelen et al, 1996), the lod-score for BRCA1 should be lower than –0.5 and/or the lod-score for BRCA2 should be higher than 0.5.

### DNA and RNA isolation

Genomic DNA was extracted from 20 ml EDTA or heparinized blood according to a standard salting out procedure (Miller et al, 1988). Total RNA was isolated from peripheral blood lymphocytes as described (Hogervorst et al, 1995).

### Reverse transcription and PCR

First-strand cDNA synthesis and PCR were performed as described (Hogervorst et al, 1995). The primer-pair used for PCR was as follows: BR2-22F 5′ TTGCGTATTGTAAGCTATTCAAAAA 3′ and BR2-25R 5′ GTCTATCCAAAACTTTATTGCCAGT 3′.

### PCR on genomic DNA and PTT

Five overlapping genomic DNA fragments of BRCA2 exon 11 and one fragment of exon 10 were amplified for use in the protein truncation test. Primer pairs used for amplification are listed in Table 1. Fifty to 100 ng genomic DNA was amplified in a 25 μl reaction volume containing buffer no. 6 (Stratagene), 5 pmol of each primer, 0.1 mM dNTP mix (final concentration), and 0.15 μl Amplitaq (Perkin-Elmer). The samples were cycled at 94°C for 45 s, 55°C for 1 min and 72°C for 2.5 min for 35 cycles. In vitro transcription and translation was performed as described elsewhere using the T7 coupled reticulo lysate system (Promega) (Hogervorst et al, 1995).

### Single-strand conformation polymorphism

The conditions for single-strand conformation polymorphism (SSCP) analysis and the primers used have been described previously (Lancaster et al, 1996).

### Cloning of BRCA2 cDNA-probes

Oligo-dT primed cDNA, obtained using total RNA isolated from the breast cancer cell line MCF7, was used as template in a nested PCR to amplify fragments of BRCA2 exon 2–9, 12–17, 18–22, and 23–27. Primer-pairs used for PCR are available upon request. Reaction conditions and thermocycling were essentially as those described in PCR and PTT. After Qiaquick column purification (Qiagen), the PCR products were ligated in a pGEM-T-Easy Vector System I (Promega) according to the suppliers protocol. One microlitre of the ligation product was transformed in XL1-Blue

**Table 2** Families carrying a BRCA2 mutation

| Family | Nucleotide | Exon | Codon | Amino acid change | Number of breast cancer cases | Number of ovarian cancer cases | a priori chance BRCA1[a] | a priori chance BRCA2[a] |
|---|---|---|---|---|---|---|---|---|
| RUL059 | 4542delC | 11 | 1438 | stop 1447 | 3 | – | ? | ? |
| EUR014 | 4708insA | 11 | 1494 | stop 1513 | 5 | 2 | 0.88 | 0.12 |
| RUL001[b] | 5579insA | 11 | 1784 | stop 1786 | 10 | – | 0.19 | 0.66 |
| RUL103 | 5579insA | 11 | 1784 | stop 1786 | 6 | 2 | 0.88 | 0.12 |
| EUR035 | 5579insA | 11 | 1784 | stop 1786 | – | 6 | 0.88 | 0.12 |
| RUL017 | 5873C>A | 11 | 1882 | stop 1882 | 7 | 2 | 0.88 | 0.12 |
| RUL024 | 8295T>A | 18 | 2689 | stop 2689 | 6 | – | 0.19 | 0.66 |
| RUL009 | 9345G>A | 23 | 3039 | splice | 9 | 1 | 0.69 | 0.19 |

[a]According to Ford et al (1998); [b]In RUL001 breast cancer cases are the total of paternal and maternal families.

© 2000 Cancer Research Campaign

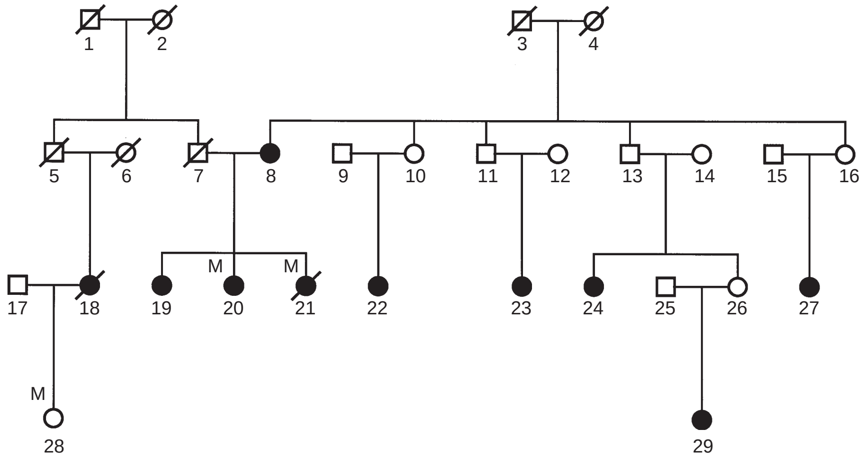

**Figure 1** Breast cancer family RUL001 carrying the 5579insA mutation in BRCA2. The mutation (indicated with M) was initially found in individual 20. One of her sisters (ID21) did carry the mutation as well, while her mother (ID8) and several of the maternal nieces do not carry the 5573insA mutation. Detection of the 5579insA mutation in the granddaughter (ID28) of the paternal uncle of the proband demonstrated the paternal origin of the mutation competent cells using electroporation. Plasmid DNA was isolated using a plasmid mini kit (Qiagen) and checked for the length of the insert by *Eco*RI digestion. Fragments of BRCA2 exon 2–9, 12–17, 18–22 and 23–27 were amplified from the clones with internal primers, and their identity was checked for by restriction analysis. Probes were $^{32}$P-labelled using a Megaprime kit (Amersham).

### Southern blotting

Five micrograms of genomic DNA was digested with either *Eco*RI or *Bgl*II, electrophoresed, blotted, and hybridized with either of the four BRCA2 probes as described earlier for BRCA1 (Petrij-Bosch et al, 1997).

### Sequencing

Samples showing an extra band of lower molecular weight in the PTT were subjected to direct sequencing using M13-tailed and biotinylated primers on a Pharmacia A.L.F. sequencer.

### RESULTS

A total of 81 families were analysed for the presence of truncating mutations in exons 10 and 11 of BRCA2, using the protein truncation test. Four different mutations were detected in six families (Table 2). Three families, RUL001, RUL103 and EUR035, carried the same mutation (5579insA). Analysis of the haplotypes of these families (Neuhausen et al, 1998) suggested that they share a common ancestor. Yet, the phenotypes of these three families differ strikingly in terms of breast and ovarian cancer incidence. EUR035 is an ovarian cancer-one family with six cases of ovarian cancer. RUL103 is a typical breast-ovarian cancer family with six breast and two ovarian cancer cases, and RUL001 is a breast cancer-only family with ten cases of breast cancer. In EUR035 and RUL103, carrier status for the 5579insA was proven for two breast cancer patients and six ovarian cancer patients. Unexpectedly, however, we found that only two breast cancer cases of RUL001 were carrying the detected mutation (ID20 and ID21), whereas the other six cases which could be tested for the mutation were negative (Figure 1). These six cases all occur in the maternal family of

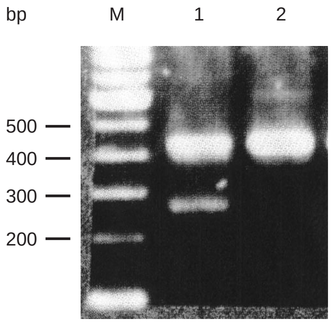

**Figure 2** RT-PCR analysis of mRNA from a 9345G>A mutation carrier in family RUL009. cDNA was amplified with a forward primer in exon 22 and a reverse primer in exon 25 of BRCA2. Lane 1: mutation carrier, lane 2: healthy unrelated control. The mutation carrier shows, in addition to the wild-type fragment of 407 bp, a band of about 250 bp. Sequence analysis revealed a deletion of the complete 164 bp of exon 23

the carrier. Subsequently, we were able to show that she inherited the mutation from her father, because we detected the mutation in the grand-daughter of her paternal uncle. One of the six mutation-negative cases (ID29, Figure 1) was selected for further testing, and was negative in the PTT.

Linkage analysis was performed previously on 13 of the 81 families selected for this study. Only four of these 13 families showed a lod-score higher than one, corresponding to a posterior probability of being linked to BRCA2 of more than 0.80 (Peelen et al, 1996). These four families remained negative in our PTT. Therefore, probands from these families were subjected to SSCP analysis of the entire BRCA2 coding region. Two of these showed an aberrant banding pattern. Sequencing of the variants revealed one premature stop-codon in exon 18 and a potential splice-donor site mutation in exon 23 in families RUL024 and RUL009 respectively (Table 2). The effect of the splice-site variant on mRNA-processing was confirmed in a breast cancer patient of RUL009 by performing reverse transcription (RT)-PCR using a forward primer in exon 22 and a reverse primer in exon 25 of BRCA2. This revealed an extra band of about 250 bp in addition to the expected wild-type product of 407 bp (Figure 2). Sequence analysis of the junction fragment revealed that 164 bp were deleted relative to the

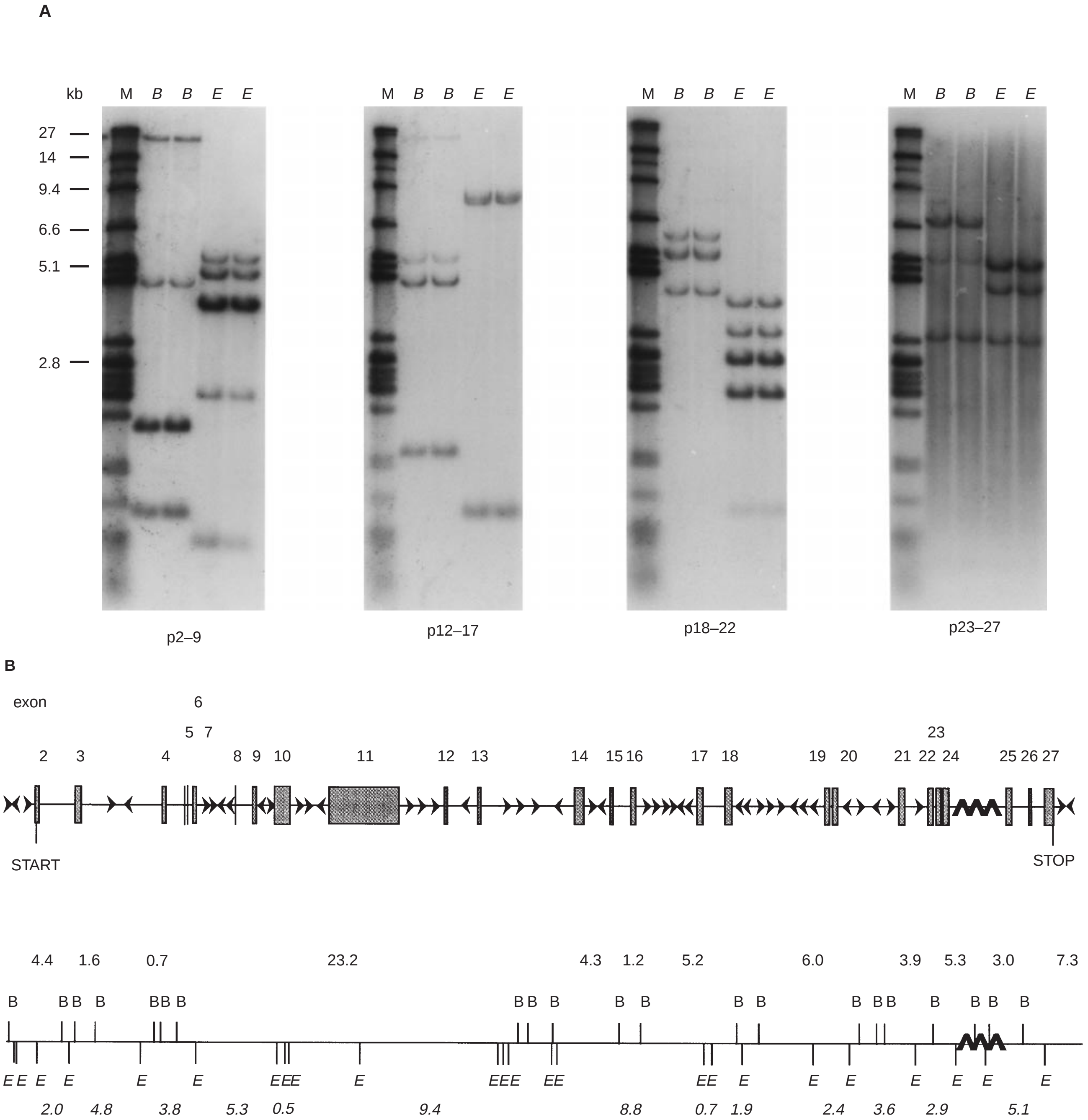

**Figure 3** (**A**) Southern blot analysis. Genomic DNA was digested with BglII (B) or *Eco*RI (E) and hybridized with either of the four cDNA probes containing exons 2–9, 12–17, 18–22 and 23–27 respectively. Hybridization patterns were as expected from the restriction maps for both BglII and *Eco*RI. (**B**) The genomic organization of BRCA2 and restriction maps of BglII (**B**) and *Eco*RI (E) as predicted by the genomic sequences from Genbank (Z74739, exons 1–24, and Z73359, exons 25–27). Numbers refer to the size of restriction fragments (in kb) detected by the four cDNA probes, above the bar for BglII and below the bar for *Eco*RI. Arrowheads indicate the presence and orientation of Alu-elements wild-type sequence, which exactly matched the complete exon 23. The 8295T>A in exon 18 created a *Dde*I-site, while the 9345G>A abolished a *Msp*I-site. Restriction-analysis with these enzymes of the respective PCR-products generated from probands in the remaining study group ($n = 73$) did not reveal additional families carrying either mutation.

Meanwhile, Southern analysis of BRCA1 had revealed that in our study population of 81 families, probands of 15 families were carriers of either the exon 13 or exon 22 deletion in BRCA1 (Petrij-Bosch et al, 1997). This prompted us to examine the entire coding region of BRCA2 by Southern analysis as well, in the remaining 58 families. Therefore, the genomic DNA of the youngest breast or ovarian cancer case from each family was digested with either *Eco*RI or *Bgl*II, blotted and hybridized with either of the four cDNA probes, containing exons 2–9, 12–17, 18–22 and 23–27 of BRCA2 respectively (Figure 3A). No alterations in the hybridization patterns, relative to controls and predicted by the restriction-map (Figure 3B), were found.

 © 2000 Cancer Research Campaign

### DISCUSSION

We have performed BRCA2 mutation analysis in 81 Dutch breast–ovarian cancer families by a combination of PTT, SSCP and Southern blot analysis. A total of eight truncating mutations were detected. Of the 81 families studied, 57 comply with the criteria used by Ford et al (1998), i.e. they have at least four cases of breast cancer and any number of ovarian cancer cases. If we apply the prior probabilities, as estimated for each of the different phenotype classes (Ford et al, 1998), that each family in this set is due to BRCA1 or BRCA2, than we would predict 24 BRCA1 and 16 BRCA2 mutations to be present among them. Indeed, while the work presented here was going on, 15 of the 81 families were identified to be due to a large genomic deletion in BRCA1 (Petrij-Bosch et al, 1997). Our PTT for exons 10 and 11 of BRCA2 would be able to detect ~70% of the mutations currently held in the BIC, which would equate to 12 mutations. Thus our finding of six truncating mutations in this region suggests that we are still missing a significant proportion of BRCA2 mutations, if the BIC mutation spectrum would apply to the Dutch population. Nevertheless, our finding that eight out of 66 BRCA1-negative families (12%) are BRCA2-positive, corresponds well with that of other large studies (i.e. >25 families) in which the entire coding region of BRCA2 was investigated (Phelan et al, 1996; Ramus et al, 1997; Serova-Sinilnikova et al, 1997; Vehmanen et al, 1997).

An obvious explanation for our results is the presence of unknown mutations that cannot be picked up by the PTT as described here, which is PCR-based and restricted to exons 10 and 11. The estimates of Ford et al (1998), as well as the BIC mutation spectrum are derived from a collection of families with a broad geographic origin and might not apply to local populations. For example, inherited breast cancer in Iceland is almost completely due to a single mutation in BRCA2, in contrast to what is predicted by the BCLC (Thorlacius et al, 1996). We decided therefore to investigate the possibility that large genomic rearrangements in BRCA2 existed in the Dutch population, prompted by our previous finding that two such deletions represent major founder mutations in BRCA1 (Petrij-Bosch et al, 1997). Southern analysis using cDNA-probes covering the entire coding region of BRCA2 of probands from 59 families did not reveal any aberrant disease-linked restriction-patterns. This contrasts sharply with our findings, and those of others (Puget et al, 1997, 1999), for BRCA1. The genomic sequence of BRCA1 contains a relatively high percentage of Alu-elements (41.5%, or one per 0.65 kb) (Smith et al, 1996). These repetitive elements have been suggested to facilitate intragenic recombination leading to exon deletions or duplications in BRCA1 (Petrij-Bosch et al, 1997; Puget et al, 1999). The genomic sequence of BRCA2 harbours approximately half as many Alu-repeats as does BRCA1, but still these elements occur on average every 1.5 kb (Genbank accessions Z74739, Z73359). In theory, therefore, Alu-mediated BRCA2-rearrangements cannot be excluded, but they are likely to occur less frequently. Although no other study has as yet investigated such a large set of families by Southern analysis, this is in accord with the fact that only two genomic rearrangements in BRCA2 have been published so far. One concerns an insertion of an Alu-element into exon 22, which resulted in its alternative splicing and skipping (Miki et al, 1996). The other is a 5 kb genomic deletion of exon 3, of which it was unclear whether it was caused by Alu-recombination (Nordling et al, 1998). These findings illustrate that large genomic rearrangements in BRCA2 are indeed part of the mutation spectrum. However, given that few major founder mutations have been identified in BRCA2 thus far, their role will probably remain limited.

The six mutations we found by PTT in our families were all located between the nucleotides 3035 and 6629. This region has been indicated as ovarian cancer cluster region (OCCR) (Gayther et al, 1997). In families with mutations in this region, the ratio of the number of ovarian cancer versus breast cancer cases is significantly higher than in families with mutations outside this region. The overall ratio of ovarian versus breast cancer was 12:24 in the six families carrying a mutation located in the OCCR, while in the two families with a mutation outside the OCCR it was 1:15. Of note, of family RUL001, only the breast cancer cases contributed by the branch of the family in which the mutation was found, were counted. Although the number of families is small, this finding supports the previous report of Gayther et al (1997)

Hence the majority of families studied here have remained negative for mutations in the BRCA2 gene, even though many showed a clear predisposition to breast and/or ovarian cancer. Further work is required to investigate other mutation mechanisms, or parts of BRCA1 and BRCA2 that have not yet been mutation-screened in great detail (such as the promotor region of both genes). This will more accurately determine the mutation spectrum in Dutch breast cancer families. Finally, the families under study here are very heterogeneous in terms of number of cases of breast and/or ovarian cancer per family. It is therefore conceivable that a proportion of these families will be assigned to mutations conferring lower breast cancer risks in genes other than BRCA1 or BRCA2, or represent chance clustering of non-genetic cases.

### ACKNOWLEDGEMENTS

We wish to thank the families for their cooperation and Inge van Leeuwen for collecting blood samples from them. This work was supported by the Dutch Cancer Society (grant RUL95–1505).

### REFERENCES

Cornelis R, Vasen H, Meijers-Heijboer H, Ford D, Van Vliet M, Van Tilborg A, Cleton F, Klijn J, Menko F, Meera Khan P, Cornelisse C and Devilee P (1995) Age at diagnosis as an indicator of eligibility for BRCA1 DNA-testing in familial breast cancer. *Hum Genet* **95**: 539–544

Ford D, Easton DF, Stratton M, Narod S, Goldgar D, Devilee P, Bishop DT, Weber B, Lenoir G, Chang-Claude J, Sobol H, Teare MD, Struewing J, Arason A, Scherneck S, Peto J, Rebbeck TR, Tonin P, Neuhausen S, Barkardottir R, Eyfjord J, Lynch H, Ponder BA, Gayther SA, Birch JM, Lindblom A, Stoppa-Lyonnet D, Bignon Y, Borg A, Hamann U, Haites N, Scott RJ, Maugard CM, Vasen H, Seitz S, Cannon-Albright LA, Schofield A, Zelada-Hedman M and the Breast Cancer Linkage Consortium (1998) Genetic heterogeneity and penetrance analysis of the BRCA1 and BRCA2 genes in breast cancer families. *Am J Hum Genet* **62**: 676–689

Gayther SA, Mangion J, Russell P, Seal S, Barfoot R, Ponder BAJ, Stratton MR and Easton D (1997) Variation of risks of breast and ovarian cancer associated with different germline mutations of the BRCA2 gene. *Nat Genet* **15**: 103–105

Hogervorst F, Cornelis R, Bout M, Van Vliet M, Oosterwijk J, Olmer R, Bakker B, Klijn J, Vasen H, Meijers-Heijboer H, Menko F, Cornelisse C, Den Dunnen J, Devilee P and Van Ommen G-J (1995) Rapid detection of BRCA1 mutations by the Protein Truncation Test. *Nat Genet* **10**: 208–212

Lancaster JM, Wooster R, Mangion J, Phelan CM, Cochran C, Gumbs C, Seal S, Barfoot R, Collins N, Bignell G, Patel S, Hamoudi R, Larsson C, Wiseman RW, Berchuck A, Iglehart JD, Marks JR, Ashworth A, Stratton MR and Futreal PA (1996) BRCA2 mutations in primary breast and ovarian cancers. *Nature Genet* **13**: 238–240

Miki Y, Katagiri T, Kasumi F, Yoshimoto T and Nakamura Y (1996) Mutation analysis in the BRCA2 gene in primary breast cancers. *Nature Genet* **13**: 245–247

© 2000 Cancer Research Campaign 

Miller SA, Dykes DD and Polesky HF (1988) A simple salting out procedure for extracting DNA from human nucleated cells. *Nucleic Acids Res* **16**: 1215–1215

Neuhausen S, Gilewski T, Norton L, Tran T, McGuire P, Swensen J, Hampel H, Borgen P, Brown K, Skolnick M, Shattuck-Eidens D, Jhanwar S, Goldgar D and Offit K (1996) Recurrent *BRCA2* 6174delT mutations in Askenazi Jewish women affected by breast cancer. *Nature Genet* **13**: 126–128

Neuhausen SL, Godwin AK, Gershoni-Baruch R, Schubert E, Garber J, Stoppa-Lyonnet D, Olah E, Csokay B, Serova O, Lalloo F, Osorio A, Stratton M, Offit K, Boyd J, Caligo MA, Scott RJ, Schofield A, Teugels E, Schwab M, Cannon-Albright L, Bishop T, Easton D, Benitez J, King MC, Ponder BA, Weber B, Devilee P, Borg Å, Narod SA and Goldgar D (1998) Haplotype and phenotype analysis of nine recurrent BRCA2 mutations in 111 families: results of an international study. *Am J Hum Genet* **62**: 1381–1388

Nordling M, Karlsson P, Wahlstrom J, Engwall Y, Wallgren A and Martinsson T (1998) A large deletion disrupts the exon 3 transcription activation domain of the BRCA2 gene in a breast/ovarian cancer family. *Cancer Res* **58**: 1372–1375

Peelen T, Cornelis RC, Van Vliet M, Petrij-Bosch A, Cleton-Jansen A-M, Meijers-Heijboer H, Klijn JGM, Vasen HFA, Cornelisse CJ and Devilee P (1996) The majority of 22 Dutch high-risk breast cancer families are due to either BRCA1 or BRCA2. *Eur J Hum Genet* **4**: 225–230

Peelen T, Van Vliet M, Petrij-Bosch A, Mieremet R, Szabo C, Van den Ouweland AMW, Hogervorst F, Brohet R, Ligtenberg MJL, Teugels E, Van der Luijt R, Van der Hout AH, Gille JJP, Pals G, Jedema I, Olmer R, Van Leeuwen I, Newman B, Plandsoen M, Van der Est M, Brink G, Hageman S, Arts PJW, Bakker MM, Willems HW, Van der Looij E, Neyns B, Bonduelle M, Jansen R, Oosterwijk JC, Sijmons R, Smeets HJM, Van Asperen CJ, Meijers-Heijboer H, Klijn JGM, De Greve J, King MC, Menko FH, Brunner HG, Halley D, Van Ommen G-JB, Vasen HFA, Cornelisse CJ, Van't Veer LJ, De Knijff P, Bakker E and Devilee P (1997) A high proportion of novel mutations in BRCA1 with strong founder effects among Dutch and Belgian hereditary breast and ovarian cancer families. *Am J Hum Genet* **60**: 1041–1049

Petrij-Bosch A, Peelen T, Van Vliet M, Van Eijk R, Olmer R, Drüsedau M, Hogervorst FBL, Hageman S, Arts PJW, Ligtenberg MJL, Meijers-Heijboer H, Klijn JGM, Vasen HFA, Cornelisse CJ, Van't Veer LJ, Bakker E, Van Ommen G-JB and Devilee P (1997) BRCA1 genomic deletions are major founder mutations in Dutch breast cancer patients. *Nature Genet* **17**: 341–345

Phelan CM, Lancaster JM, Tonin P, Gumbs C, Cochran C, Carter R, Ghadirian P, Perret C, Moslehi R, Dion F, Faucher M-C, Dole K, Karimi S, Foulkes W, Lounis H, Warner E, Goss P, Anderson D, Larsson C, Narod SA and Futreal PA (1996) Mutation analysis of the *BRCA2* gene in 49 site-specific breast cancer families. *Nature Genet* **13**: 120–122

Puget N, Torchard D, Serova-Sinilnikova OM, Lynch HT, Feunteun J, Lenoir GM and Mazoyer S (1997) A 1-kb Alu-mediated germ-line deletion removing BRCA1 exon 17. *Cancer Res* **57**: 828–831

Puget N, Sinilnikova OM, Stoppa-Lyonnet D, Audoynaud C, Pag, Lynch HT, Goldgar D, Lenoir GM and Mazoyer S (1999*a*) An Alu-mediated 6-kb duplication in the BRCA1 gene: a new founder mutation? *Am J Hum Genet* **64**: 300–302

Puget N, Stoppalyonnet D, Sinilnikova OM, Pages S, Lynch HT, Lenoir GM and Mazoyer S (1999*b*) Screening for germ-line rearrangements and regulatory mutations in BRCA1 led to the identification of four new deletions. *Cancer Res* **59**: 455–461

Rahman N and Stratton MR (1998) The genetics of breast cancer susceptibility. *Annu Rev Genet* **32**: 95–121

Ramus SJ, Kotejarai Z, Friedman LS, Van der Looij M, Gayther SA, Csokay B, Ponder BA and Olah E (1997) Analysis of BRCA1 and BRCA2 mutations in Hungarian families with breast or breast-ovarian cancer. *Am J Hum Genet* **60**: 1242–1246

Serova-Sinilnikova OM, Boutrand L, Stoppa-Lyonnet D, Bressac-Depaillerets B, Dubois V, Lasset C, Janin N, Bignon YJ, Longy M, Maugard C, Lidereau R, Leroux D, Frebourg T, Mazoyer S and Lenoir GM (1997) BRCA2 mutations in hereditary breast and ovarian cancer in France. *Am J Hum Genet* **60**: 1236–1239

Smith TM, Lee MK, Szabo CI, Jerome N, Mceuen M, Taylor M, Hood L and King MC (1996) Complete genomic sequence and analysis of 117 kb of human DNA containing the gene BRCA1. *Genome Res* **6**: 1029–1049

Szabo CI and King MC (1997) Population genetics of BRCA1 and BRCA2. *Am J Hum Genet* **60**: 1013–1020

Thorlacius S, Olafsdottir G, Tryggvadottir L, Neuhausen S, Jonasson JG, Tavtigian SV, Tulinius H, Ögmundsdottir HM and Eyfjörd JE (1996) A single *BRCA2* mutation in male and female breast cancer families from Iceland with varied cancer phenotypes. *Nature Genet* **13**: 117–119

Tonin PN, Mesmasson AM, Futreal PA, Morgan K, Mahon M, Foulkes WD, Cole DEC, Provencher D, Ghadirian P and Narod SA (1998) Founder BRCA1 and BRCA2 mutations in French Canadian breast and ovarian cancer families. *Am J Hum Genet* **63**: 1341–1351

Vehmanen P, Friedman LS, Eerola H, Sarantaus L, Pyrhonen S, Ponder BAJ, Muhonen T and Nevanlinna H (1997) A low proportion of BRCA2 mutations in Finnish breast cancer families. *Am J Hum Genet* **60**: 1050–1058

*© 2000 Cancer Research Campaign*
